# Knowledge landscape of tumor-associated macrophage research: A bibliometric and visual analysis

**DOI:** 10.3389/fimmu.2023.1078705

**Published:** 2023-01-18

**Authors:** Feng Zhou, Yang Liu, Cong Liu, Fangfei Wang, Jianxiang Peng, Yong Xie, Xiaojiang Zhou

**Affiliations:** ^1^ Department of Gastroenterology, Digestive Disease Hospital, The First Affiliated Hospital of Nanchang University, Nanchang, Jiangxi, China; ^2^ Department of Gastroenterology, Gastroenterology Institute of Jiangxi Province, Nanchang, Jiangxi, China; ^3^ Key Laboratory of Digestive Diseases of Jiangxi Province, The First Affiliated Hospital of Nanchang University, Nanchang, Jiangxi, China; ^4^ Department of Gastroenterology, Jiangxi Clinical Research Center for Gastroenterology, Nanchang, Jiangxi, China

**Keywords:** tumor-associated macrophage, cancer, bibliometrics, visualization, hotspots

## Abstract

**Background and aims:**

Tumor-associated macrophage (TAM) is a highly abundant immune population in tumor microenvironment, which plays an important role in tumor growth and progression. The aim of our study was to explore the development trends and research hotspots of TAM by bibliometric method.

**Methods:**

The publications related to TAM were obtained from the Web of Science Core Collection database. Bibliometric analysis and visualization were conducted using VOSviewer, CiteSpace and R software.

**Results:**

A total of 6,405 articles published between 2001 and 2021 were included. The United States and China received the most citations, whereas the University of Milan, the university of California San Francisco and Sun Yat-sen University were the main research institutions. Mantovani, Alberto from Humanitas University was the most productive authors with the most citations. Cancer Research published the most articles and received the most co-citations. Activation, angiogenesis, breast cancer, NF-κB and endothelial growth factor were important keywords in TAM research. Among them, PD-1/L1, nanoparticle, PI3Kγ, resistance and immune microenvironment have become the focus of attention in more recent research.

**Conclusions:**

The research on TAM is rapidly evolving with active cooperation worldwide. Anticancer therapy targeting TAM is emerging and promising area of future research, especially in translational application. This may provide guidance and new insights for further research in the field of TAM.

## Introduction

Macrophages has long been considered to be an evolutionarily ancient cell type involved in tissue homeostasis and immune defense. Recently, macrophages were discovered to regulate a variety of diseases depending on the surrounding tissue microenvironment, especially for cancer (1–3). Tumor-associated macrophage (TAM) is a highly abundant immune population in tumors, which plays an important role in cancer progression, metastasis and treatment resistance.

The ability of macrophages to adapt to subtle changes in external stimuli results in the diversity of TAM between different types of cancer or within the same tumor. Macrophages are generally divided into classically activated M1 phenotypes and alternately activated M2 phenotypes to reflect the Th1/Th2 immune response. Although TAM often shows more similar patterns to M2- polarized macrophages that suppresses immune responses and promotes tumor progression, the simplified M1/M2 definition might not be sufficient to cover the full complexity of TAMs (4). In fact, TAM rarely completely follow the true M1 and M2 phenotypes, and even some macrophages can share both M1 and M2 signatures (5–7). In addition, the cell subsets do not exist at a steady stage and changes as the tumor progresses. Each population has a unique landscape based on the type, stage and immune composition of the infiltrated tumors. The plasticity and heterogeneity allow TAM to promote or suppress tumor growth and progression through multiple pathways. Therefore, there is great significance to quantitatively evaluate the research status, focus area and development trend of TAM.

Bibliometrics is an interdisciplinary science that provides a comprehensive and objective assessment of knowledge carriers by mathematics and statistics (8–10). The bibliographic analysis helps scholars understand the development of specific topic and reveals the evolution trend of this field. This study aimed to explore the landscape of tumor-associated macrophages, hoping to provide new clues and ideas for future research in the field of TAM.

## Methods

### Search strategies

Scientific output data was extracted from the Web of Science Core Collection (WoSCC) database, which is one of the most widely used source for academic and bibliometric analysis. The search formula was presented as follows: TS = (“tumor associated macrophage*”) OR (“tumor-associated macrophage*”) OR (“tumour associated macrophage*”) OR (“tumour-associated macrophage*”) OR (“cancer associated macrophage*”) OR (“cancer-associated macrophage*”). The publication period was limited to between 2001 and 2021, and the publication type was limited to original articles written in English. Moreover, we also used broader terms as a benchmark dataset to better evaluate the overall trend of immune cell research in cancer such as “tumor OR tumour OR cancer” and “T cell OR macrophage OR neutrophil*”. The literature search and data collection were performed independently by two researchers to ensure the reliability of the results.

### Data collection

Original data was extracted from selected publications, including titles, abstracts, authors, affiliations, countries/regions, journals, publication years, references and keywords. The H-index of scholars, impact factor (IF) and Journal citation reports (JCR) division of journals were obtained from the Web of Science. Productivity of activities is measured by the number of citations. Overlapping items were merged into a single element and misspelled words were corrected artificially. The cleaned data were exported for further analysis.

### Bibliometric analysis

Bibliometric indicators are used to quantitatively describe and evaluate the characteristics of literature and its trends. We used R software to conduct Lotka’s Law analysis (11). VOSviewer is a bibliometric tool for developing scientometric network and knowledge visualization (12). The network graph generated by VOSviewer displays the size of nodes according to the number of publications, where closely related nodes are grouped into the same cluster. The connection indicates the association of different nodes, and the thickness of the connection depends on the strength of the association. Centrality is used to measure the importance of a node’s location in the network, and nodes with centrality greater than 0.1 are generally considered as critical nodes. CiteSapce software provides new angle for the bursts of research hotspots in the field of TAM (13).

## Results

**Figure 1:**
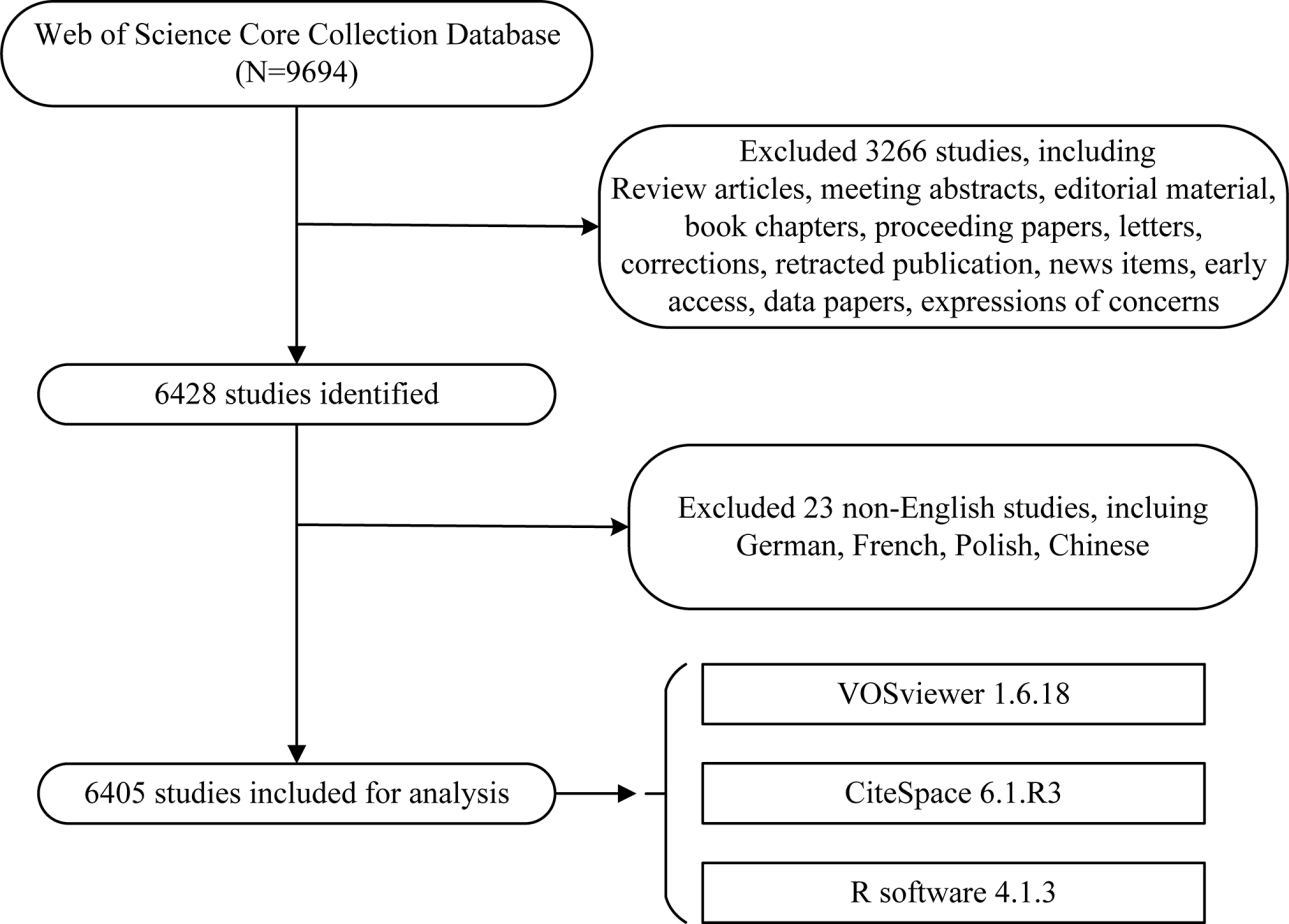
Flowchart of literature screening.

A total of 9,694 literatures were published in the field between 2001 and 2021. According to the exclusion criteria, we finally included 6,405 eligible original articles in our study. The specific flow diagram was illustrated in **Figure 1**.

### Growth trend of publication

**Figure 2:**
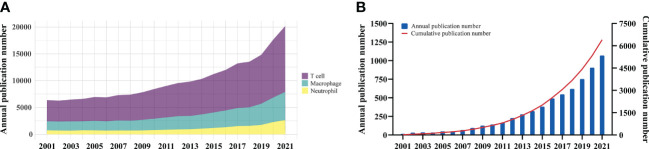
**(A)** The overall growth trend of annual immune cell research in cancer. **(B)** Annual and cumulative growth trend of TAM publications.

The overall growth trend of immune cell research in cancer were showed in **Figure 2A**. Although T cell is the most heavily studied immune population, the field of macrophage showed similar increase rate of up to three times. For tumor-associated macrophage research, the number of articles published exhibited a steady increase from 2001 to 2021 (**Figure 2B**). The output of publications from 2001 to 2008 was low, with less than 100 articles per year. With the fast increase in the number of annual publications, there were 6,028 articles on TAM published between 2009 and 2021, accounting for 94.1% in the past two decades. These findings indicated that TAM has gained great interest and entered the phase of rapid development.

### Distribution of countries/regions and institutions

**Figure 3:**
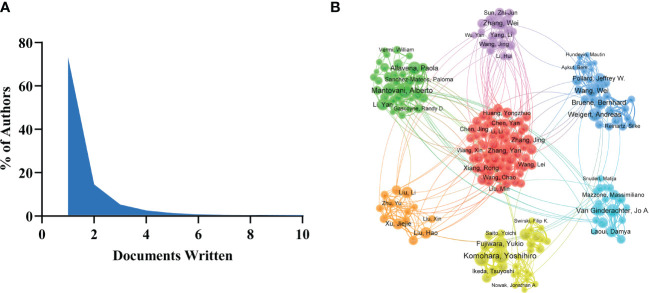
**(A)** Scientific productivity of authors based on Lotka’s Law. **(B)** The network map of authors on tumor-associated macrophage research.

The publications on TAM were conducted by 5,294 institutions in 99 countries/regions (**Table 1**). The United States received the highest citations (N=123799), followed by China (N=71126) and Italy (N=28368). Annual citations per publication peaked in the middle of the study period in most countries/regions, especially for Italy (**Figure 3A**). Although China carried out the most publications, the average number of citations is lower than other countries/regions. The bibliometric map revealed the tight communications between countries/regions (**Figure 3B**). Intense collaborations between countries/regions resulted in thicker connecting lines between nodes. Of them, the centrality of the United States is as high as 0.37, suggesting that it plays a strong bridge role between the cooperations. In addition, China, Italy, Germany, Japan and United Kingdom are also important nodes among clusters, with centrality greater than 0.1.

**Table 1:** The top 10 countries/regions and institutions that have contributed to publications on tumor-associated macrophage research.

Country	Centrality	Count	Citation	Institution	Centrality	Count	Citation
United States	0.37	1947	123799	Univ Milan	0.12	67	12892
China	0.13	2157	71126	Univ Calif San Francisco	0.02	55	8971
Italy	0.14	369	28368	Sun Yat Sen Univ	0.05	165	7727
Germany	0.20	469	25772	Univ Texas Md Anderson Canc Ctr	0.12	107	6741
Japan	0.17	599	22314	Harvard Univ	0.08	55	6529
United Kingdom	0.15	253	15545	Mem Sloan Kettering Canc Ctr	0.11	73	6436
France	0.05	199	13833	Fudan Univ	0.01	152	6425
Switzerland	0.06	135	11200	Shanghai Jiao Tong Univ	0.07	149	6397
Netherlands	0.06	170	10305	Stanford Univ	0.04	74	6335
Spain	0.04	157	9849	Massachusetts Gen Hosp	0.04	55	5708

The 5,294 institutions constituted seven main clusters (**Figure 3C**). The University of Milan, the university of California San Francisco and Sun Yat-sen University were the most productive institutions, with centrality ranged from 0.02 to 0.12. The University of Texas MD Anderson Cancer Center and Memorial Sloan-Kettering Cancer Center also had a centrality of more than 0.1 and belonged to a key node of the network.

### Author and co-author analysis

**Figure 4:**
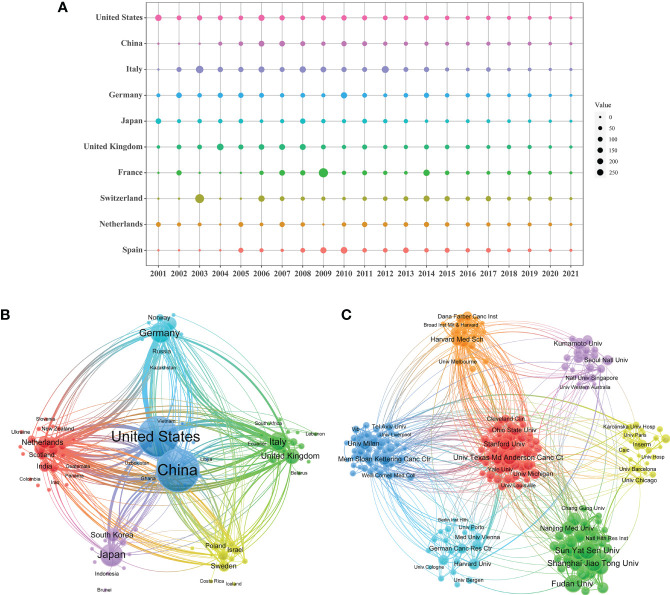
**(A)** Annual citations per publication for the top 10 countries. The network map of countries/regions **(B)** and institutions **(C)** on tumor-associated macrophage research.

There were 41,399 authors involved in the study of tumor-associated macrophages. Scientific productivity based on Lotka’s law shows that 73.1% of authors contributed only one publication (**Figure 4A**). Mantovani, Alberto from Humanitas University received the most citations (N=10675) with the most publications (**Table 2**). The next productive authors were Sica, Antonio from University of Eastern Piedmont Amedeo Avogadro (N=9344) and Coussens, Lisa M from University of California San Francisco (N=5745). There were active collaborations among the author clusters of seven different colors (**Figure 4B**). A certain degree of collaborations existed between two linked nodes in different clusters, such as Pollard, Jeffrey W and De Palma, Michele.

**Table 2:** The top 10 productive authors and cited authors in the field of tumor-associated macrophages.

Authors	Count	H-index	Cited author	Count	H-index
Mantovani, Alberto	39	182	Mantovani, Alberto	10675	182
Komohara, Yoshihiro	36	45	Sica, Antonio	9344	72
Fujiwara, Yukio	33	32	Coussens, Lisa M	5745	81
Bruene, Bernhard	30	73	DeNardo, David G	4519	40
Weigert, Andreas	27	40	Lawrence, Toby	3909	40
Sica, Antonio	26	72	Ruffell, brian	3848	29
Aiba, Setsuya	24	40	Van Ginderachter, Jo A	3702	62
Takeya, Motohiro	23	62	Weissleder, Ralph	3337	168
Van Ginderachter, Jo A	22	45	Pollard, Jeffrey W	3246	30
Van Ginderachter, Jo A	22	62	Pittet, Mikael	3187	72

### Journals and cited academic journals

**Figure 5:**
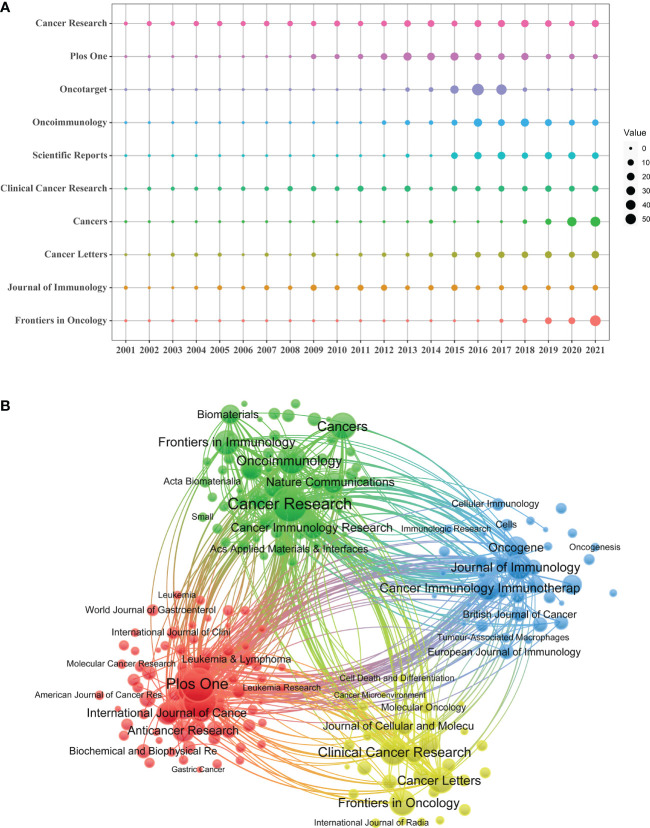
**(A)** Annual number of publications for the top 10 journals. **(B)** The network map of journals on tumor-associated macrophage research.

A total of 1,201 journals were identified in this research field. The journal with the most publications was Cancer Research (N=173), followed by Plos One (N=152) and Oncotarget (N=148). Among the top ten journals related to TAM, 7 journals have an impact factor greater than 5, and 5 journals were at the Q1 JCR division (**Table 3**). At the same time, Cancer Research generated the most co-citations (N=18479). **Figure 5A** showed Scientific Reports, Cancers and Frontiers in Oncology were relatively new to this field, but developed rapidly.

**Table 3:** The top 10 journals and cited journals related to tumor associated macrophages.

Journal	Count	IF (2021)	JCR (2021)	Cited journal	Citation	IF (2021)	JCR (2021)
Cancer Research	173	13.312	Q1	Cancer Research	18479	13.312	Q1
Plos One	152	3.752	Q2	Cancer Cell	9039	38.585	Q1
Oncotarget	148	–	–	Clinical Cancer Research	8991	13.801	Q1
Oncoimmunology	110	7.723	Q1	Proceedings of the National Academy of Sciences of the United States of America	8035	12.779	Q1
Scientific Reports	104	4.996	Q2	Plos One	7910	3.752	Q2
Clinical Cancer Research	95	13.801	Q1	Journal of Experimental Medicine	7358	17.579	Q1
Cancers	90	6.575	Q1	Journal of Immunology	8030	5.426	Q2
Cancer Letters	86	9.756	Q1	Journal of Clinical Investigation	6982	19.456	Q1
Journal of Immunology	85	5.426	Q2	Blood	6437	25.476	Q1
Frontiers in Oncology	83	5.738	Q2	Nature Communication	3943	17.694	Q1

The cited journals network indicated the association between two journals. Journals are divided into four clusters, and the size of nodes represented the number of co-citations (**Figure 5B**). There was similar theme between journals of the same color, especially for red cluster.

### Keywords co-occurrence, clusters and bursts

Keywords were extracted from the 6,405 published articles. As shown in **Table 4**, NF-κB (N=336), endothelial growth factor (N=204) and PD-L1 (N=170) were the most commonly involved molecules. Activation (N=1002), polarization (N=903) and angiogenesis (N=806) appeared more frequently for pathological processes. As for specific diseases, breast cancer (N=840), colorectal cancer (N=287) and lung cancer (N=251) received the most attention.

**Table 4:** The top 10 molecules, pathological process and disease related to tumor associated macrophages research.

Molecules	Count	Pathological processes	Count	Diseases	Count
NF-Kappa B	336	Activation	1002	Breast cancer	840
Endothelial growth factor	204	Polarization	903	Colorectal cancer	287
PD-L1	170	Angiogenesis	806	Lung cancer	251
CD163	149	Infiltration	341	Hepatocellular carcinoma	240
Colony stimulating factor	146	Differentiation	291	Glioblastoma	164
INF-gamma	141	Proliferation	255	Gastric cancer	164
TGF-β	118	Epithelial mesenchymal transition	219	Ovarian cancer	148
STAT3	116	Apoptosis	206	Pancreatic cancer	141
Nitric Oxide	107	Metabolism	99	Melanoma	137
CD68	93	Recruitment	97	Prostate cancer	136

**Figure 6:**
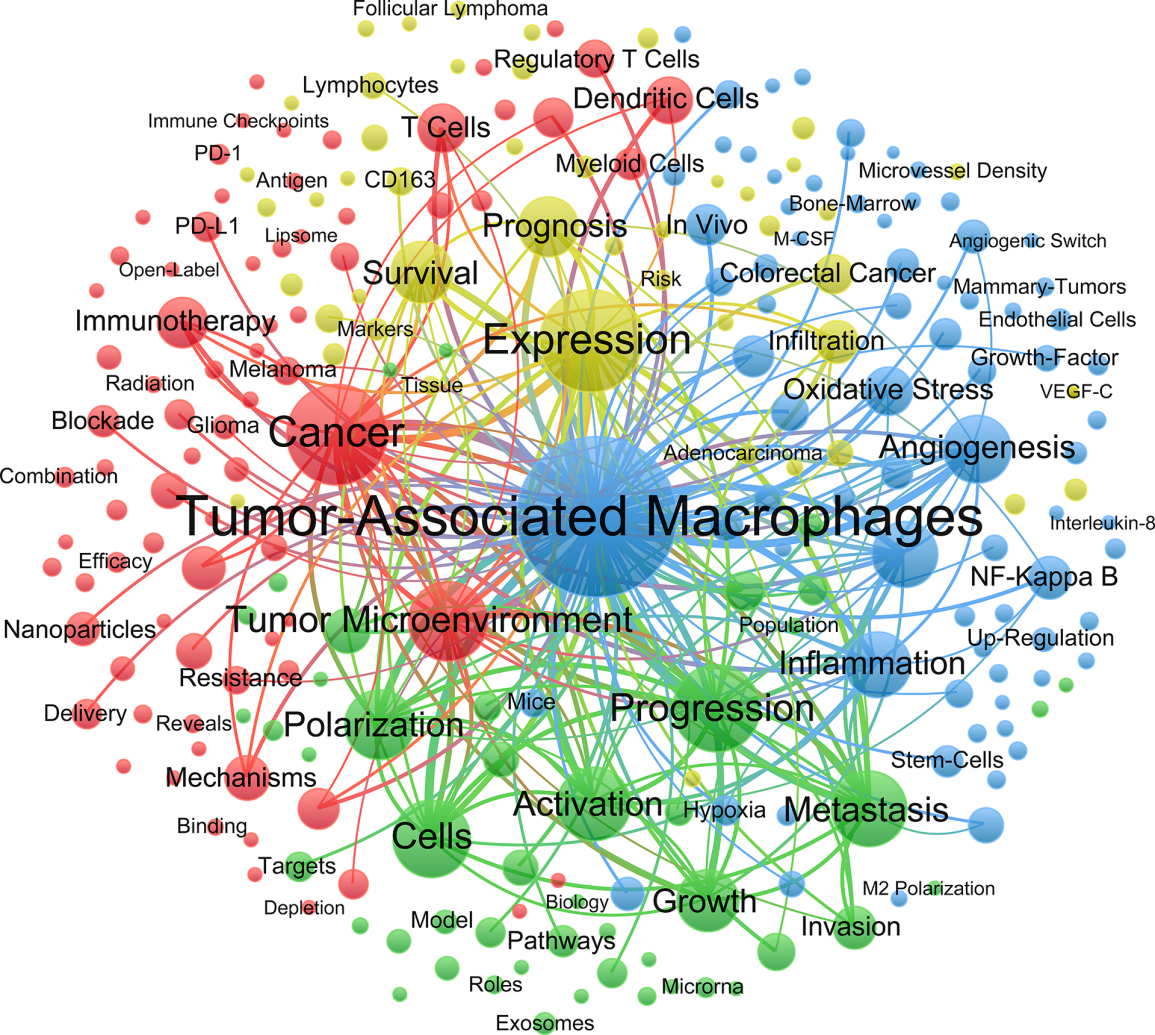
The network map of keywords on tumor-associated macrophage research.

Clustering keywords help to identify the distribution of research content on a specific topic (**Figure 6**). The largest blue cluster consisted of keywords was associated with the pathological processes and molecules of macrophages, including angiogenesis, NF-Kappa B and oxidative stress. Red cluster involved the cancer treatment, including immunotherapy, resistance and nanoparticle. Yellow cluster mainly explored the factors associated with tumor prognosis.

**Figure 7:**
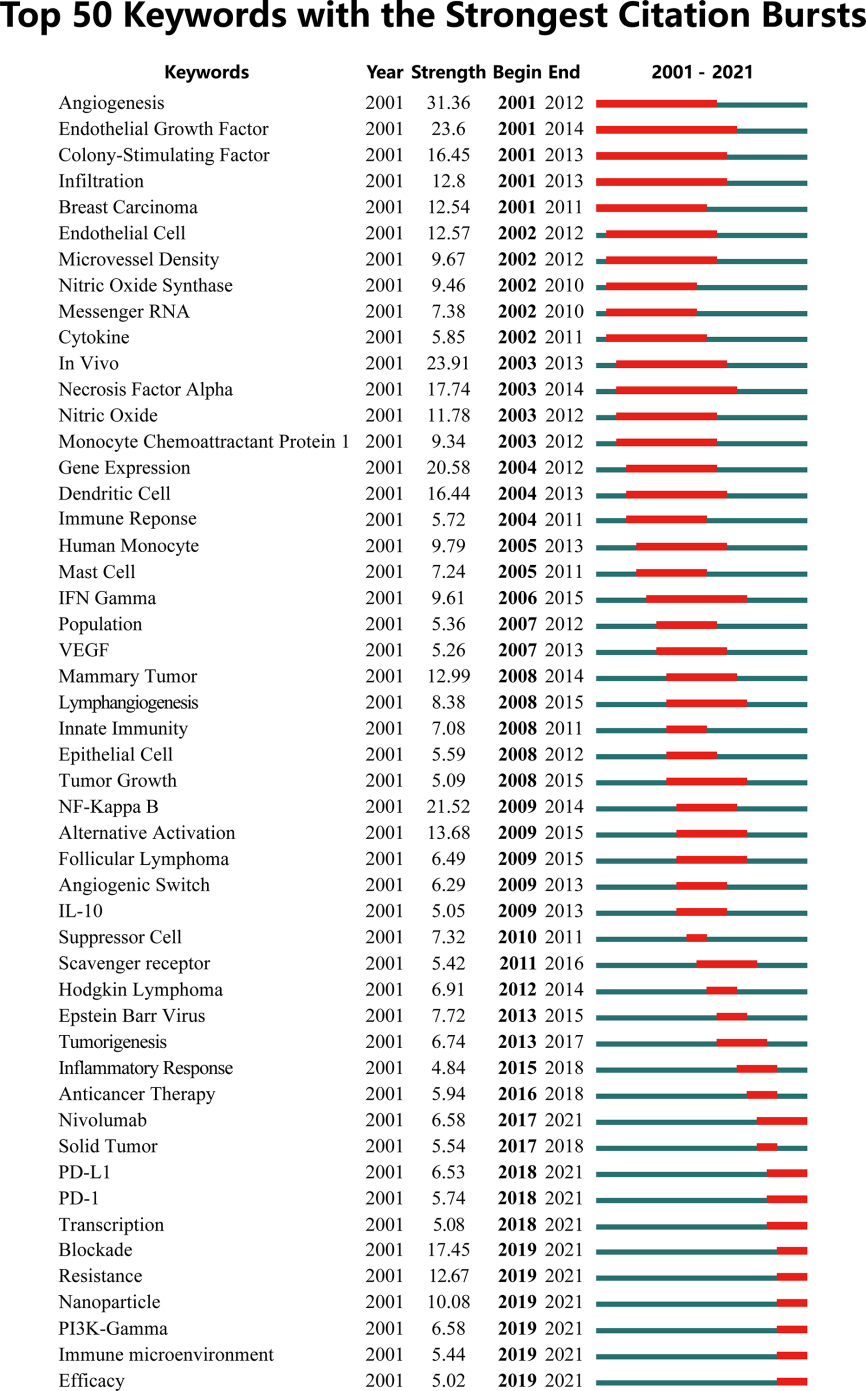
The top 50 keywords with the strongest citation bursts on tumor-associated macrophage research.

A visual map was constructed to show the trend of keywords bursts, where the red part represented the duration of citation burst (**Figure 7**). The early burst keywords included angiogenesis, epithelial growth factor, and colony stimulating factor. Citation bursts in the middle period (2011-2016) were significantly attenuated with a decrease in hotspot keywords such as NF-κB, Hodgkin lymphoma and scavenger receptor. In recent years (2018-2020), the treatment of cancer received increasing attention from researchers. PD-1/L1, PI3Kγ, resistance, nanoparticle and immune microenvironment has become the focus of attention of current research.

## Discussion

Tumor-associated macrophage is an important part of the tumor microenvironment and interacts with cancer cells to maintain the most of characteristics of tumors. The diversity of TAM forms a complex communication network between cancer and immune cells (14). In this study, we extracted TAM studies from public databases for bibliometric analysis to identify its hotspots and development trends. The increasing trend in annual publication volume demonstrated the significant potential of TAM in cancers.

The United States and China were the countries with the most citations. The distribution of institutions is consistent with countries/regions based on geographical location. However, the average citations for most countries/regions and institutions did not correspond well to the number of publications in this field. More robust efforts may be required to deeply clarify the role and mechanism of TAM in tumor. Meanwhile, the United States achieves a maximum level of cooperation in TAM research with a centrality of 0.37. Compared with other countries, it constitutes several cooperative subnetworks to better promote the development of the field, such as the University of Texas MD Anderson Cancer Center, Memorial Sloan-Kettering Cancer Center and University of Chicago.

Regarding the productivity of authors, Alberto Mantovani received the most citations in the TAM field. Mantovani mainly focused on the regulatory effect of chemokines on TAM (15–17) and some related anticancer drugs such as trabectedin (18, 19). Given the limitation of the binary M1-M2 classification of macrophage, Mantovani also attempted to divide macrophages into additional subsets (M2a, M2b and M2c) (20) or used looser terms (M1-like and M2-like) (21). Besides the collaborations with Mantovani, Antonio Sica made efforts to link inflammatory reaction to cancer through NF-κB (22–24). Coussens’s group from University of California San Francisco focused more on the immune cell crosstalk in breast cancer (25–27).

Cancer research published the most articles and received the highest number of co-citations. Scientific Reports, Cancers and Frontiers in Oncology were emerging journals spreading macrophage research. Papers published in highly cited journals such as Nature, Blood and Cell were more likely to be reviewed by scholars and have more access to citations.

The clustering analysis of keywords indicated that TAM research ranged from the biological properties of macrophages to the targeted therapy of cancer. TAM is highly related to specific pathological context, and its complex mechanism in tumors has attracted extensive attention. Angiogenesis is the initial research focus, which provides basic condition for tumor growth and dissemination. Angiogenesis is the initial research focus, which provides basic condition for tumor progression. Studies have shown that TAM can promote angiogenesis through the release of cytokines, growth factors and matrix metalloproteinases or the expression of TIE2 receptors (28–32). NF-κB is considered to be a molecular link between the inflammation and cancer. In the middle period, it gradually presented the highest citation burst strength. NF-κB activation in macrophages is essential for tumor growth. Inhibition of IKKβ leads to a significant reduction in tumor onset and load of several inflammation-induced cancer models (33–35). However, TAM often shows alternative immunosuppressive M2-like phenotype, which is not easily reconciled with the proinflammatory function of NF-κB in TAM. The scavenger receptor MARCO expressed on the surface of macrophages is able to regulate macrophage polarization and enhance tumor killing (36, 37).

Recently, anticancer therapy targeting TAM has generated the most research enthusiasm. Immunosuppression microenvironment limits the efficacy of checkpoint block and adoptive cell therapy, particularly in solid tumors (38). TAMs can suppress immunotherapy efficacy by inhibiting T-cell activity and enhancing the expression of PD-L1 in the TME. In addition to inhibiting T cell activation, a study from Sydney et al. showed that immune checkpoint inhibitor PD-1/L1 also inhibited TAM phagocytosis, which may be associated with M2 polarization (39). In-depth inquiry of PD-1/L1 expanded the knowledge of PD-1/L1 from its role in T cells to many other cell types, including macrophages. PI3K/Akt is also an important signaling pathway participating in macrophages survival, proliferation and cytoskeleton rearrangement. PI3K induces TAMs into M2-like phenotype and is closely correlated with poor clinical outcomes of cancers (40). Inhibition of PI3Kγ make tumors sensitive to immune checkpoint inhibitors by reprogramming TAM, demonstrating the importance of macrophage-mediated immune microenvironment for optimal immunotherapy efficacy (41–43). CSF-1R expressed on TAMs is involved in the activation of PI3K signaling pathway, and regulate the immune inhibition in macrophages. Blockade of CSF1 has been shown to deplete TAM and prevent TAM recruitment to the tumor (44, 45). Targeting TAM can play its unique regulatory function in promoting the antitumor effects of current immunotherapy.

Due to the unique biophysical properties, nanoparticles show greater advantages and potentials in cancer treatment. Compared with traditional drugs, nanoparticles can extend retention time and achieve targeted delivery with a decreased toxicity. Some studies have reported that nanoparticles specifically enhance anticancer immune responses by targeting TAM (46–50). The rich blood circulation and strong phagocytosis ability also make macrophages themselves become the optimal carrier of drug delivery. TAM allows the delivery of nanotherapeutic drugs to tumor cells and alters the spatial diffusion of drugs within the tumor (51, 52). Imaging the response between tumors and nanomaterials provide a reliable basis for the development of highly effective targeted therapies.

The bibliometric study reflected the development trend and research hotspots in this field to a certain extent. At the same time, this study has several limitations. The included literatures were collected from WOSCC database, which caused the omission of some information. Furthermore, there were potential biases in bibliometric method based on natural language processing. Excessive adjustments for inaccurate elements may reduce the credibility of the results.

In conclusion, the research on TAM is rapidly evolving with active cooperation worldwide. And anticancer therapy targeting TAM is emerging and promising area of future research, especially in translational application. This may provide guidance and new insights for further research in the field of TAM.

## Data availability statement

The raw data supporting the conclusions of this article will be made available by the authors, without undue reservation.

## Author contributions

XZ and YX designed the study. YL, CL and FW conducted data extraction. FZ, YX and JP performed data analysis. FZ drafted the manuscript. XZ interpreted the data and revised the manuscript. All authors contributed to the article and approved the submitted version.
